# Editorial: Lipid signaling in immunity: from inflammation and ageing to cancer and therapeutic opportunities

**DOI:** 10.3389/fimmu.2026.1992695

**Published:** 2026-09-30

**Authors:** Juan Pablo Rodríguez, Mauricio César De Marzi, Jesús Balsinde

**Affiliations:** 1 Laboratorio de Investigaciones Bioquímicas de la Facultad de Medicina (LIBIM), Instituto de Química Básica y Aplicada del Nordeste Argentino (IQUIBA-NEA), Universidad Nacional del Nordeste, Consejo Nacional de Investigaciones Científicas y Técnicas (UNNE-CONICET), Corrientes, Argentina; 2 CONICET-Universidad Nacional de Luján, Instituto de Ecología y Desarrollo Sustentable (INEDES), Grupo de Investigaciones Básicas y Aplicadas en Inmunología y Bioactivos (GIBAIB), Buenos Aires, Argentina; 3 Universidad Nacional de Lujan, Departamento de Ciencias Básicas, Laboratorio de Inmunología, Buenos Aires, Argentina; 4 Instituto de Biología y Genética Molecular, Consejo Superior de Investigaciones Científicas UVa, Valladolid, Spain; 5 Centro de Investigación Biomédica en Red de Diabetes y Enfermedades Metabólicas Asociadas (CIBERDEM), Instituto de Salud Carlos III, Madrid, Spain

**Keywords:** lipid channeling, lysophospholipid mediators, phospholipase A_2_, tissue homeostasis, tumor immunosuppression

Lipids are no longer viewed merely as membrane components or energy stores, but as dynamic regulators of immune cell activation, adaptation, and fate. The articles assembled in this Research Topic extend this view by converging on a central principle: the immunological effects of lipids are determined not by abundance alone, but by molecular identity, metabolic routing, cellular source, tissue localization, and timing. In this sense, context is not simply a modifier of lipid signaling; it is part of the mechanism. This principle emerges across the four biological settings represented in the collection—macrophage metabolism, liver injury, tumor-associated immune dysfunction, and inflammatory skin disease—where lipid pathways can preserve homeostasis or promote pathology depending on when and where they operate (**Figure 1**).

**Figure 1:**
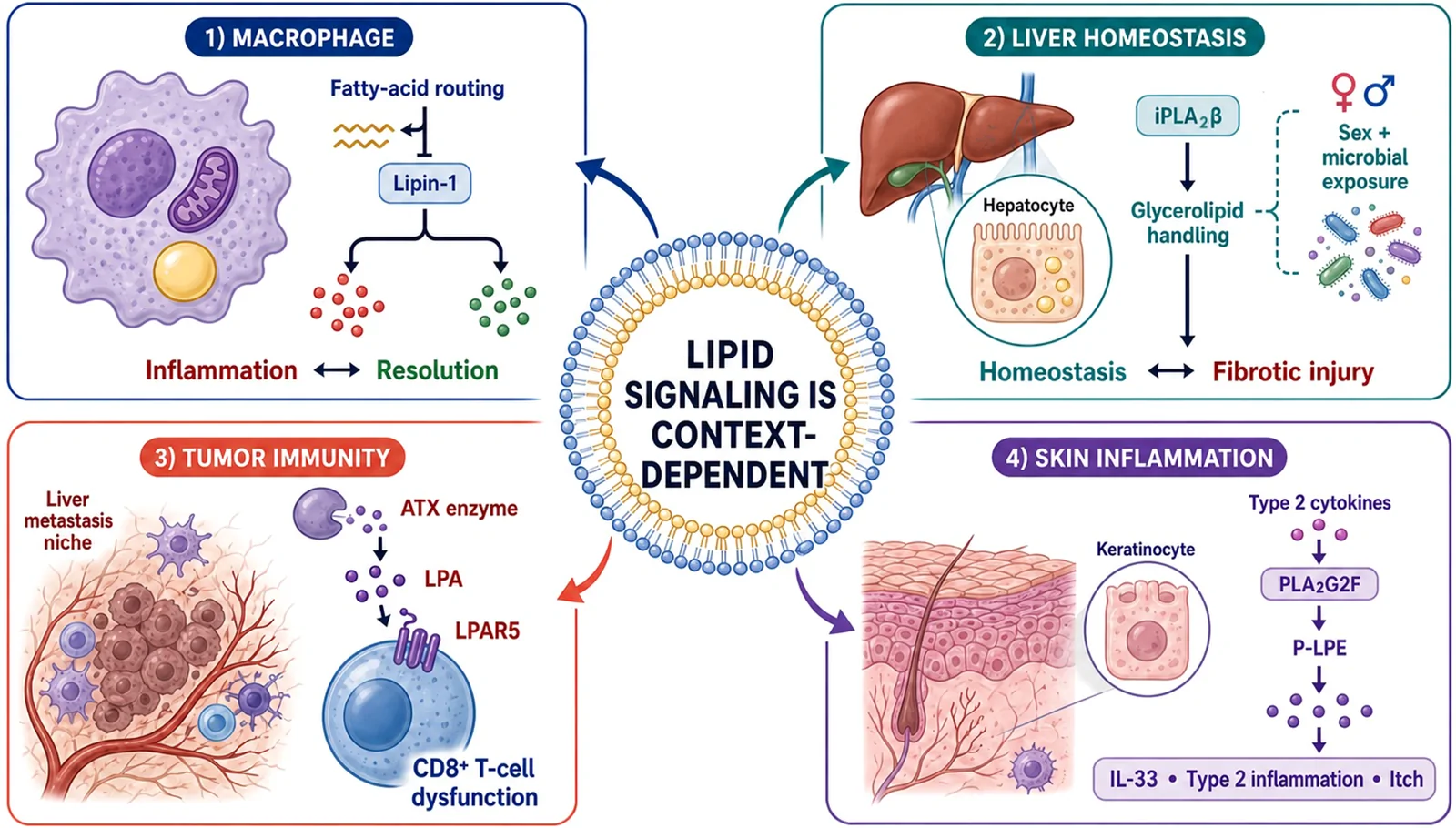
Lipid signaling in immunity. Schematic representation of the four complementary settings explored in this Research Topic. (1) In macrophages, fatty-acid routing and the multifunctional regulator lipin-1 influence the balance between inflammatory activation and resolution. (2) In the liver, iPLA_2_β contributes to glycerolipid homeostasis, while sex and microbial exposure modify the transition from adaptation to fibrotic injury. (3) In the metastatic liver niche, the secreted enzyme autotaxin (ATX) generates lysophosphatidic acid (LPA), which signals through LPAR5 and can promote CD8^+^ T-cell dysfunction. (4) In the epidermis, type 2 cytokines induce PLA_2_G2F-dependent production of lysoplasmenylethanolamine (P-LPE), linking keratinocyte lipid metabolism with IL-33 expression, type 2 inflammation, and itch. Collectively, these studies illustrate how the immunological outcome of lipid signaling is determined by lipid identity, metabolic routing, cellular source, tissue location, and timing.

Bamgbose et al. provide a broad conceptual framework for understanding these context-dependent effects. Their review examines how macrophages channel fatty acids toward mitochondrial β-oxidation, glycerolipid synthesis and lipid-droplet storage, sphingolipid production, or polyunsaturated fatty acid-derived mediators. These routes support distinct and sometimes opposing outcomes, including inflammatory activation, efferocytosis, resolution of inflammation, metabolic adaptation, and lipotoxic dysfunction. Lipin-1 illustrates this complexity particularly well. Positioned at a branch point between phosphatidic acid and diacylglycerol metabolism, and also acting through non-enzymatic regulatory functions, lipin-1 can amplify inflammatory signaling in lipid-loaded macrophages while supporting mitochondrial fitness and efferocytosis in pro-resolving states. The relevant therapeutic question is therefore not whether a pathway is intrinsically inflammatory, but how lipid flux is organized in a defined macrophage population and tissue niche.

The study by Xu et al. reinforces this principle through a model in which loss of group VIA calcium-independent phospholipase A_2_ (iPLA_2_β/PLA_2_G6) interacts with environmental exposure, age, and sex. In mice naturally exposed to pathogens, global PLA_2_G6 deficiency aggravated hepatocellular apoptosis, inflammatory fibrosis, and susceptibility to endotoxin-induced injury in both sexes. However, defective hepatic glycerolipid synthesis, reduced triacylglycerol and lipoprotein secretion, and reduced adiposity were most evident in males. These findings reveal a homeostatic role for iPLA_2_β and caution against interpreting lipid-metabolizing enzymes as uniformly pathogenic targets. They also emphasize that sex and microbial environment can uncover phenotypes that remain muted under specific-pathogen-free conditions. More broadly, the work argues for experimental designs that integrate lipid metabolism with biological variables often treated as secondary.

Two additional contributions demonstrate how locally generated lipids can organize pathological immune niches. Turner et al. investigate the autotaxin–lysophosphatidic acid (ATX–LPA) axis in liver metastases from uveal melanoma, a disease characterized by hepatic tropism and limited responsiveness to conventional immunotherapy. Autotaxin is a secreted ectoenzyme that generates extracellular LPA, an immunosuppressive lipid mediator that can limit cytotoxic CD8^+^ T-cell function through LPAR5. In this setting, the authors show that LPA induces low-level but persistent ERK phosphorylation in effector CD8^+^ T cells, with dynamics distinct from canonical T-cell receptor signaling, and that loss of LPAR5 improves CD8^+^ T-cell survival and cytokine function during chronic stimulation. Together with the detection of elevated ATX transcripts in tumor-associated hepatic CD8^+^ T cells, these findings identify ATX–LPA signaling as a plausible contributor to immune dysfunction in the metastatic liver. The study also highlights the potential of combining pathway-selective immune intervention with liver-directed cytoreduction such as percutaneous hepatic perfusion with melphalan, while appropriately leaving the efficacy and optimal sequencing of such combinations for future investigation.

Miki et al. extend the theme to the epidermis by identifying a PLA_2_G2F–lysoplasmalogen axis that links keratinocyte lipid metabolism with type 2 inflammation and itch in atopic dermatitis. PLA_2_G2F, induced by type 2 cytokines, generates lysoplasmenylethanolamine (P-LPE). Genetic deletion of Pla2g2f reduced keratinocyte IL-33 expression, epidermal hyperplasia, serum IgE, type 2 inflammation, and scratching in mice. Pharmacological inhibition or enzymatic degradation of P-LPE also suppressed itch, whereas exogenous P-LPE partially restored scratching in deficient animals. Importantly, P-LPE was elevated in the stratum corneum of patients and correlated with disease severity. This work therefore connects a defined lipid product to epithelial alarm signaling, a clinically relevant symptom, and a potential biomarker, while illustrating the advantages of tissue-localized therapeutic intervention.

Viewed together, these studies reveal lipid signaling as a spatially organized network that connects intracellular metabolic decisions with communication between immune and structural cells. They also expose a recurring translational challenge. Broad inhibition of lipid synthesis or lipid-metabolizing enzymes may disrupt protective buffering, membrane homeostasis, efferocytosis, or tissue repair. Greater therapeutic precision will require discrimination among enzymatic and non-enzymatic functions, lipid species, receptors, cellular sources, and disease stages. Strategies such as lymphocyte-selective LPAR5 inhibition, topical targeting of PLA_2_G2F, macrophage-directed modulation of lipid-routing nodes, or interventions informed by sex and microbial exposure exemplify this shift from pathway suppression to context-specific control.

The next phase of this field should combine quantitative lipidomics with metabolic-flux analysis, spatial and single-cell approaches, and genetic models that resolve cell type and tissue compartment. Longitudinal studies will also be necessary to determine when adaptive lipid handling becomes maladaptive during ageing, chronic inflammation, and cancer progression. By bringing together mechanistic and translational perspectives across liver, skin, macrophages, and T cells, this Research Topic underscores that the biological meaning of a lipid signal cannot be separated from its location and context. Understanding this lipid geography will be essential for converting pathway-level insight into selective biomarkers and therapies. We thank all authors, reviewers, and the editorial team for their contributions to this Research Topic.

